# Serum concentrations of thyroid hormones, cholesterol and triglyceride, and their correlations together in clinically healthy camels (*Camelus dromedarius*): Effects of season, sex and age 

**Published:** 2013

**Authors:** Javad Tajik, Alireza Sazmand, Seyedhossein Hekmati moghaddam, Aria Rasooli

**Affiliations:** 1 *Department of Clinical Studies, School of Veterinary Medicine, Shahid Bahonar University of Kerman, Kerman, Iran; *; 2 *Department of Agriculture, Payame Noor University, Yazd, Iran; *; 3 *Department of Laboratory Sciences, Faculty of Paramedicine, Shahid Sadoughi University of Medical Sciences, Yazd, Iran;*; 4 *Department of Clinical Studies, School of Veterinary Medicine; Shahid Chamran University of Ahvaz, Ahvaz, Iran.*

**Keywords:** *Camelus dromedaries*, Cholesterol, Thyroid hormones, Triglyceride

## Abstract

To evaluate the effects of season, sex and age on serum concentrations of thyroid hormones, cholesterol and triglyceride, and their correlations together in dromedarian camels, these parameters were measured in 180 clinically healthy dromedary camels. No significant difference was detected for the measured serum parameters between the two sexes and among the different age groups of camels and none of them had significant correlation with the age of the animals. There was a significant correlation between serum T_4_ and triglyceride (r = -0.243, *p *= 0.002). There were significant differences between summer and winter seasons in the serum concentrations of T_4_ (*p *< 0.001), T_3_ (*p *= 0.01) and triglyceride (*p* < 0.001). In winter, the serum concentration of triglyceride had a significant correlation with the age of the sampled camels (r = -0.235, *p *= 0.026). In male camels,T_4_ had a marginally significant correlation with cholesterol (r= -0.158, *p *= 0.06).The effects of season, sex and age on the serum concentrations and relationships between thyroid hormones, cholesterol and triglyceride in dromedary camels can be proposed as the probable causes of the controversial findings in the previous studies.

## Introduction

Thyroid hormones are known as important modulators of general metabolism.^1^ These hormones regulate energy metabolism in which carbohydrates and lipids are the major constituents.^2^ Thyroid hormones affect lipid metabolism by increasing lipolysis in adipose tissue and stimulating lipogenesis by increasing the activities of some enzymes.^3^ The serum cholesterol level generally changes inversely with thyroid activity,^4^^,^^5^ however, there are some contradictory findings regarding the relationship of serum thyroid hormones with cholesterol and triglycerides in camels. Some studies showed that the serum concentration of thyroid hormones were not related to cholesterol levels in male camels,^6^ however, Nazifi *et al*. found a significant positive correlation between serum thyroid hormones and cholesterol in male dromedary camels.^7^

There are controversial findings regarding age related changes in the serum concentrations of cholesterol and triglyceride in domestic animals. Also, seasonal variations in the serum concentration of thyroid hormones have been reported in some domestic animals.^8^^-^^10^ However, the effects of these variations on serum concentrations of cholesterol and triglyceride and their correlations with thyroid hormones in different species are not completely known.^11^

Dromedary camel occupies a unique place among all domesticated animals due to its tolerance of heat stress. There is little known about the serum lipids and their relationship with thyroid hormones in this species and to the best of our knowledge, there has been no previous study about the effects of season, age and sex on their relationships in dromedary camels. Therefore, this study was undertaken to investigate the effects of season, age and sex on the serum profiles and the relationships between these parameters in dromedarian camels.

## Materials and Methods

The research was carried out in Yazd province, a semi-arid region in the center of Iran and clinically healthy one-humped camels which were kept by local farmers and fed low quality diets containing mainly straw, barely and wilted grass were sampled. The clinical examination included evaluation of body temperature, mucous membranes, skin, and cardiorespiratory, gastrointestinal and nervous systems. The study was performed from July to September 2011 (summer months) and January to February 2012 (winter months) and 90 randomly selected camels were sampled in each sampling period.

All samples were taken in the morning. After clinical examination, jugular blood samples in plane tubes, free from anticoagulant, were collected. Camels were of both sexes, with different ages and were selected randomly. The age of the animals was estimated using dental characteristics.^12^

The blood serum was separated after centrifugation at 750 *g* for 15 min and the serum samples were stored at -20 ˚C until analysis. Samples showing gross hemolysis were discarded.

Serum total protein was measured by Biuret method, cholesterol by enzymatic colorimetric method (Cholesterol oxidase - phenol + aminophenazone), and triglyceride by enzymatic colorimetric method (Glycerol-3-phosphate oxidase - phenol + aminophenazone) of McGowan *et al*., using a spectrophotometer (Model AA200; Shimadzu, Tokyo, Japan).^13^^,^^14^ Estimation of triiodothyronine (T_3_) and thyroxine (T_4_) levels in serum were made using sandwich ELISA method (Diaplus Inc., San Francisco, USA).

Statistical analysis was performed using SPSS (Version 12.0; SPSS, Chicago, USA). Two sample *t*-test was used to detect differences in the parameters between the two sexes and between two seasons. Correlations were analyzed by Pearson’s correlation tests and analysis of variance (ANOVA) tests were used to compare the serum concentrations of the measured factors between the different age groups. Differences were considered significant at *p* < 0.05.

## Results

Overall, 146 male camels and 34 female camels aged three months to 18 years old were sampled. The average ages (Mean ± SEM) of the male and female camels were 7.26 ± 0.24 and 8.32 ± 0.84 years, respectively. The average age of both sexes had no significant difference. The average temperatures of summer and winter in the sampling region were 30.76 and 8.63 ˚C, respectively.

The results of the measurement of serum concentrations of thyroid hormones, total protein, cholesterol and triglyceride in different sexes and age groups of dromedary camels are shown in Table 1.

There was no significant difference between the male and female camels in the serum concentrations of thyroid hormones, total protein, cholesterol and triglyceride. None of the measured serum parameters had a significant correlation with the age of the sampled camels. There was a significant correlation between serum T_4_ and triglyceride (r = -0.243, *p* = 0.002) and serum concentration of triglyceride had a significant correlation with cholesterol (r = 0.209, *p* = 0.005).

When sampling seasons were evaluated separately, the same significant relationships between the measured factors were seen. However, in winter, the serum concentration of triglyceride had a significant correlation with the age of the sampled camels (r = -0.235, *p* = 0.026). There were significant differences between the summer and winter seasons in the serum concentrations of T_4_ (*p* < 0.001), T_3_ (*p* = 0.01) and triglyceride (*p* < 0.001). The results of the measured serum concentrations of thyroid hormones, total protein, cholesterol and triglyceride in different seasons are shown in Table 2.

**Table 1:** The concentrations (Mean ± SEM) of serum thyroid hormones, total protein, cholesterol and triglyceride in clinically healthy camels in different sexes and age groups

	**Number ** **of camels**	**Cholesterol** **(mmol L** ^-1^ **)**	**Triglyceride** **(mmol L** ^-1^ **)**	**Total protein** **(g dL** ^-1^ **)**	**Triiodothyronine** **(nmol L** ^-1^ **)**	**Thyroxine** **(nmol L** ^-1^ **)**
**All samples**	180	0.86 ± 0.03	0.37 ± 0.04	7.39 ± 0.05	3.55 ± 0.16	171.90 ± 0.70
**Male **	146	0.88 ± 0.03	0.37 ± 0.04	7.41 ± 0.05	3.53 ± 0.17	171.20 ± 0.76
**Female **	34	0.78 ± 0.06	0.35 ± 0.05	7.25 ± 0.17	3.65 ± 0.42	175.80 ± 0.76
**G1 **	48	0.78 ± 0.04	0.32 ± 0.04	7.30 ± 0.11	3.63 ± 0.29	183.50 ± 1.40
**G2 **	109	0.91 ± 0.04	0.36 ± 0.04	7.48 ± 0.06	3.59 ± 0.22	172.30 ± 0.89
**G3 **	23	0.86 ± 0.07	0.51 ± 0.08	7.15 ± 0.15	3.20 ± 0.39	145.40 ± 1.80

G1 (≤ 2 years); G2 (2 years < and ≤ 10 years); G3 (10 years <).

**Table 2:** The concentrations (Mean ± SEM) of serum thyroid hormones, total protein, cholesterol and triglyceride in clinically healthy camels in different seasons and sexes

		**Number ** **of camels**	**Cholesterol** **(mmol L** ^-1^ **)**	**Triglyceride** * **(mmol L** ^-1^ **)**	**Total protein** **(g dL** ^-1^ **)**	**Triiodothyronine** * **(nmol L** ^-1^ **)**	**Thyroxine** * **(nmol L** ^-1^ **)**
**All samples**		180	0.86 ± 0.03	0.38 ± 0.04	7.39 ± 0.05	3.55 ± 0.16	171.90 ± 7.08
**Summer**	**Total **	90	0.90 ± 0.03	0.54 ± 0.07	7.32 ± 0.08	3.00 ± 0.22	115.90 ± 6.80
	**Male **	71	0.92 ± 0.04	0.55 ± 0.08	7.40 ± 0.08	2.80 ± 0.21	115.70 ± 7.20
	**Female**	19	0.72 ± 0.07	0.49 ± 0.09	6.70 ± 0.28	4.20 ± 0.79	117.60 ± 21.10
**Winter**	**Total **	90	0.83 ± 0.04	0.19 ± 0.01	7.46 ± 0.06	4.02 ± 0.21	219.20 ± 9.10
	**Male **	75	0.82 ± 0.04	0.18 ± 0.01	7.42 ± 0.07	4.17 ± 0.24	219.40 ± 0.99
	**Female**	15	0.82 ± 0.09	0.24 ± 0.03	7.65 ± 0.16	3.25 ± 0.46	218.50 ± 2.36

* Asterisk indicates significant differences between two seasons (*p* < 0.05).

The camels were divided into three groups, according to their age as G_1_ ≤ 2 years, 2 years < G_2_ ≤ 10 years, and G_3 _> 10 years. There were no significant differences between the three age groups for the serum concentrations of thyroid hormones, total protein, cholesterol and triglyceride (*p* > 0.05). Separate evaluation of age groups showed no significant change in evaluated relationships.

Both sexes were evaluated separately. In the male camels, T_4 _had a significant correlation with triglyceride (r = -0.235, *p* = 0.005) and had a marginally significant correlation with cholesterol (r= -0.158, p=0.06). Also, the age had a marginally significant correlation with triglyceride (r = 0.151, *p* = 0.06). In the female camels T_4_ had a significant correlation with triglyceride (r = -0.424, *p* = 0.031). 

## Discussion

 Although there are some previous studies about the serum concentrations of thyroid hormones, cholesterol and triglyceride in dromedary camels, there is little information about the effects of season, sex and age on their relationships.

The concentrations of the measured serum thyroid hormones, cholesterol and triglyceride in the current study were somewhat different from the previously reported ranges for Iranian dromedary camel.^7^ Nazifi *et al. *measured serum concentrations of thyroid hormones, cholesterol and triglyceride in 30 male dromedary camels in summer, in which the serum concentrations of T_4_, cholesterol and triglyceride were rather equal to the results of this study.^7^ However, serum concentration of T_3_ in the current study was higher than that reported by Nazifi *et al*. (3.55 ± 0.16 vs. 2.57 ± 0.04, respectively).^7^ Nazifi *et al. *measured the serum concentrations of cholesterol and triglyceride in male dromedary camels. According to our results, in male camels the serum concentration of cholesterol was the same and serum triglyceride was less than that reported by Nazifi *et al*. (0.53 to 0.90 mmol L^-1^ in different age groups).^15^

Our results showed that serum T_4_ had a negative correlation with triglyceride, and in male camels had a marginally significant correlation with cholesterol. There are some contradictory findings regarding the relation of the serum thyroid hormones with the cholesterol and triglyceride levels. It is believed that serum cholesterol level generally varies inversely with thyroid activity. The net effect of thyroid hormones on the cholesterol metabolism is to increase the rate of cholesterol catabolism by liver.^4^^,^^5^^,^^16^

Other studies, however, showed no significant correlation between serum thyroid hormones and cholesterol levels in camels, male goats and fat tailed sheep.^6^^,^^16^^,^^18^ On the other hand, Nazifi *et al.* found significant positive correlations of serum thyroxin with cholesterol and triglyceride levels in clinically healthy male camels. They believed that these discrepancies may be due to the hydration or health status of the animals. Our results showed that other factors such as sex may affect these relationships.^7^

In the current study, no significant differences for serum thyroid hormones and triglyceride were found between the sexes. Similar to our results, sex had no significant effect on the serum thyroid hormones, cholesterol and triglyceride in Turkoman horses, sheep and water buffaloes.^3^^,^^19^^,^^20^ However, a significant difference between sexes concerning the plasma cholesterol level in some sheep breeds and concerning the thyroid hormones in some breeds of pig has been reported.^3^

We found no significant difference between the different age groups in serum concentrations of thyroid hormones, cholesterol and triglyceride and there was no significant correlation between the measured factors and age. Similar results regarding the thyroid hormones, cholesterol and triglyceride were found in water buffalo and goat.^20^^,^^21^ In the current study, separate evaluation of both sexes showed a significant correlation of age with triglyceride in male animals, which was same as that reported in water buffalo.^20^

An age related increase in serum concentrations of cholesterol and triglyceride in male dromedary camel and in Turkoman horses has been reported,^15^^,^^19^ while age related decrease in their serum concentrations has been reported in sheep.^3^ In calves, the cholesterol concentration increased transiently with age, but triglycerides showed no consistent change.^22^

Serum levels of thyroid hormones are mainly affected by general body metabolism, season and water availability.^7^ Camel is known to be a seasonal breeder and the period of maximum breeding activity for the males is winter and the spring seasons. Zia-ur-Rahman *et al*. reported that the serum concentrations of thyroid hormones in non-rutting one-humped male camels are higher than rutting camels.^23^ According to our results, serum concentrations of thyroid hormones in winter were higher than summer. In contrast to our results, Nazifi *et al*. showed that serum concentration of thyroid hormones in dromedary camel in summer was higher than winter.^7^ On the other hand, serum concentrations of T_3_ and T_4_ in goat and dog are higher in winter than in summer and it is believed that a cold environment increases the secretion of thyrotrophic hormone, which results in a higher serum concentration of thyroid hormones.^8^^,^^9^ Yagil *et al*. reported that camel thyroid was inhibited in summer due to dehydration. This inhibition assists in the preservation of body water by decreasing pulmonary water loss and reducing the basic metabolism.^10^ Similarly, Khanna *et al*. reported that during the summer, T_4_ levels fell gradually in dehydrated dromedary camels and increased after rehydration, whereas in the winter, T_4_ levels increased in dehydrated camels.^24^ Our results showed no significant difference in serum total protein between the different seasons, however, the mean serum total protein of camels in the current study was higher than that of Nazifi *et al*. study (Mean: 7.39 g dL^-1^ vs. 5.09 to 6.74 g dL^-1^, respectively).^11^ Since serum total protein increases in dehydrated animals, probable dehydration of sampled camels in the current study may be the reason for these controversial findings. It seems that the hydration status of animals should be considered in evaluation of seasonal changes of thyroid hormones in camel. 

According to our results, the serum concentration of triglyceride was significantly higher in summer than winter but the serum cholesterol had no significant difference between the two seasons. Similar to our results, Amin *et al*. reported serum triglyceride increment during the dry season in one-humped camels.^25^ El-Bahrawey and Hassanein reported serum cholesterol decrement in male dromedary camels in summer.^26^ In Holstein heifers, the serum concentration of cholesterol in winter was higher than in summer and environmental temperature had significant correlations with serum T_3_ and cholesterol.^27^ It is generally believed that thyroid hormones affect cholesterol metabolism,^4^^,^^5^^,^^16^ and observed seasonal change in serum cholesterol may be partly due to changes in thyroid hormones.

The cause of these findings and some contradictory findings regarding the relation between serum thyroid hormones, triglyceride and cholesterol are not clear and may be due to the effect of some factors such as breed, geography and diet on serum profiles of sampled groups.

However, the results of the current study showed the age, sex and season as the probable causes of the controversial findings in the previous studies.
